# Application Characteristics of Ultra-Fine 15 μm Stainless Steel Wires: Microstructures, Electrical Fatigue, and Ball Formation Mechanisms

**DOI:** 10.3390/mi16030326

**Published:** 2025-03-12

**Authors:** Hsiang-Chi Yang, Fei-Yi Hung, Bo-Ding Wu, Yi-Tze Chang

**Affiliations:** Department of Materials Science and Engineering, National Cheng Kung University, Tainan 70101, Taiwan; n56134011@gs.ncku.edu.tw (H.-C.Y.); n58071065@gs.ncku.edu.tw (B.-D.W.);

**Keywords:** stainless steel wire, mechanical properties, electrical fatigue, electrify annealing, wire bonding

## Abstract

Stainless steel wires exhibit excellent mechanical properties and are widely used in engineering applications. This study fabricates 15 μm stainless steel wires for potential integration into wire bonding technology for electronic packaging. The research explores the microstructural characteristics, electrical conduction mechanisms, and ball formation behavior of ultra-fine stainless-steel wires to assess their feasibility for wire bonding applications. Results indicate that both 15 μm and 30 μm stainless steel wires exhibit elongated grains with outstanding tensile strength and hardness. Compared to the 30 μm wires, the 15 μm wires undergo more pronounced work hardening, leading to higher tensile strength and resistance. This study investigates the differences between vacuum and electrified annealing processes to address the work hardening and ductility issues in stainless steel wires. Results confirm that the hardness of the original wire significantly decreases after vacuum annealing at 780 °C for 15 min. Furthermore, using the derived equation, $T=\frac{IV}{2.3085\times 10^{-3}}+25$, the annealing temperature of 780 °C is converted into an equivalent current, and electrify annealing is conducted under a condition of 0.08 A for 15 min. The annealed wires exhibit a softening effect and enhance ductility. Furthermore, due to stored deformation energy and recrystallization effects, the electrical fatigue life of 15 μm stainless steel wires is approximately 300 cycles. After electrifying annealing, the base microstructure becomes more homogeneous due to thermal effects, reducing fatigue life to around 150 cycles. However, due to the softening effect, the annealed wires make the EFO process easier and minimize solidification segregation in the free air ball (FAB) microstructure, demonstrating their potential for electronic packaging applications.

## 1. Introduction

Electronic packaging is a critical aspect of the semiconductor industry, enabling the connection between processed chips and external contacts for functionality. The three commonly used bonding techniques are wire bonding, automated tape bonding, and flip-chip bonding [1]. Among these, wire bonding offers advantages such as lower cost, a simple and efficient process, and well-established technology. In wire bonding, the bonding wire transmits electrical signals from the chip while dissipating heat generated during operation. Therefore, it must exhibit excellent electrical and thermal conductivity. Common bonding wire materials include gold (Au), silver (Ag), and copper (Cu). Gold and silver wires provide high conductivity, thermal performance, and corrosion resistance [2,3]; however, their high cost and rapid formation of intermetallic compounds (IMCs) with aluminum substrates during bonding present challenges. Copper wires, while offering superior electrical and thermal conductivity with slower IMC formation than gold and silver [4], suffer from poor corrosion and oxidation resistance. During packaging, copper wires can react with oxygen in the air or halogen ions in the encapsulation material, leading to oxidation or intergranular corrosion, which degrades conductivity and mechanical strength [5]. To address these limitations, this study explores the application of stainless-steel wires in wire bonding, leveraging their superior corrosion resistance and cost-effectiveness.

The 304 stainless steel is an austenitic stainless steel with no magnetic properties. It is the most commonly used food-grade stainless steel and is widely applied in industrial manufacturing, interior decoration, and kitchenware [6]. The alloy primarily consists of 18 wt.% chromium and 8 wt.% nickel, earning it the designation of 18-8 stainless steel. Known for its excellent corrosion resistance and balanced mechanical properties, 304 stainless steel is versatile for various applications [7].

Considering that the strength and hardness of 304 stainless steel wires significantly increase after cold drawing [8,9,10], their reliability in wire bonding applications is reduced. To achieve wire softening and toughness, this study utilized 304L stainless steel, which has a lower carbon, nitrogen, hydrogen, and oxygen content compared to 304 stainless steel. The 304L stainless steel exhibits lower mechanical strength but superior corrosion resistance, making it well suited for applications requiring both corrosion resistance and bonding ability [11]. Subsequently, vacuum annealing conditions were optimized to determine the ideal temperature and holding time. The annealing parameters were then converted into an equivalent electrical current for electrified annealing, utilizing electrically induced recrystallization to enhance ductility [12]. Furthermore, to accurately reflect the operational conditions of semiconductor components, this study analyzed the microstructure and mechanical properties of stainless-steel wires, established their electrical characteristics and electrical fatigue mechanisms using DC, and evaluated their discharge ball formation capability. The results demonstrate that 304L stainless steel fine wires exhibit electrically induced recrystallization, offering not only excellent mechanical strength and electrical fatigue life but also the ability to form FAB, meeting the requirements for wire bonding applications. To date, commercially available stainless-steel wires have diameters exceeding 50 µm, whereas this study successfully fabricated 15 µm wires (the thinnest reported globally), demonstrating a significant technological advancement. Moreover, no prior research has been found applying stainless steel wire in wire bonding, making this study highly innovative. The findings provide valuable insights and a potential reference for next-generation packaging technologies.

## 2. Experimental Procedures

**Figure 1:**
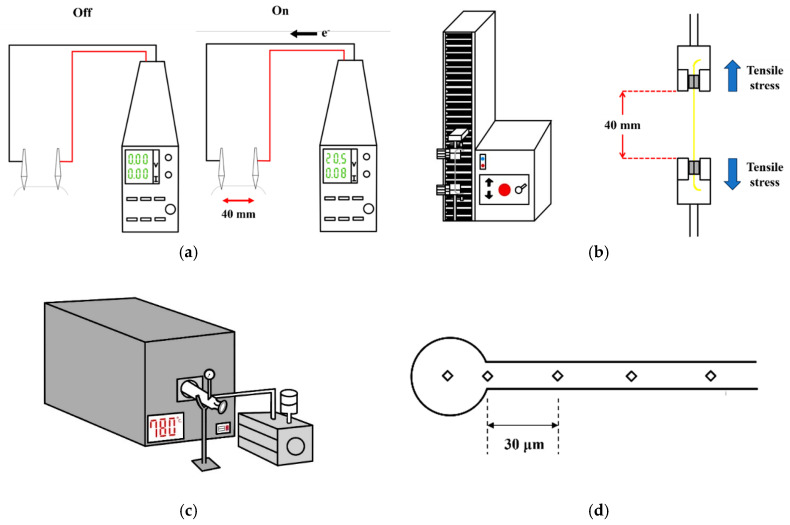
Schematic of experimental procedures: (**a**) I-V curve measurement and EFM test; (**b**) micro-tensile testing machine and tensile test; (**c**) vacuum furnace; and (**d**) hardness measurements in HAZ and FAB.

In this study, 304L stainless steel fine wires (C ≤ 0.02 wt.%, N ≤ 300 ppm, H ≤ 3 ppm, O ≤ 50 ppm) with radii of 15 µm (15SS/ cold reduction: 38%) and 30 µm (30SS/ cold reduction: 36%) were used, both derived from 304L stainless steel thick wire material through a cold drawing process. The drawing speed was 80 m per second. The microstructure of the wires was analyzed using an optical microscope (OM, OLYMPUS BX41M-LED, Tokyo, Japan), while hardness was measured with a Vickers hardness tester (HV, SHIMADZU HMV-G31S, Kyoto, Japan). Tensile properties were evaluated using a micro-tensile testing machine. A DC power supply machine assessed the I-V characteristics, electrical resistance, and electrical fatigue mechanism (EFM). Figure 1a illustrates the measurement setup for obtaining I-V curves and conducting electrical fatigue tests using the DC power supply machine, with the length fixed at 40 mm. During I-V curve measurements, the interval of the current increment was 0.02 A until the wire began glowing red, after which the interval was reduced to 0.01 A. The dynamic resistance was calculated by applying Ohm’s law to the linear region of the I-V curve.

The electrical fatigue test was conducted using two measurement parameters. The first approach tested each 304L stainless steel fine wire at 80% of its fuse current. In the second approach, the test was standardized using the 80% fuse current of the 15SS wire (0.08 A). Each electrical fatigue cycle consisted of a 60 s ON phase followed by a 5 s OFF phase, with the number of cycles recorded until wire fracture. As shown in Figure 1b, the tensile testing setup utilized a micro-tensile testing machine with a fixed wire length of 40 mm and a strain rate of 0.005 s^−1^. The stress–strain curve was measured to determine strength and elongation.

Figure 1c illustrates the setup for vacuum annealing. The annealing parameters included holding at 780 °C for 15, 30, 45, and 60 min, as well as at 480 °C for 12 h. The literature indicates that the reverse transformation of martensite in austenitic stainless steel occurs at approximately 500 °C, accompanied by a decrease in hardness. Therefore, these parameters were selected for vacuum furnace annealing to examine potential differences in martensite content [13]. After analyzing the microstructure and measuring hardness, X-ray diffraction (XRD) analysis was used to examine potential phase transformations before and after vacuum annealing, which could influence the hardness. Based on these findings, the optimal annealing condition was 780 °C for 15 min. Using the equation, $T=\frac{IV}{2.3085\times 10^{-3}}+25$ [14], this condition was converted into an equivalent electrify annealing process at 0.08 A for 15 min. The study further analyzed the differences in microstructure, hardness, electrical properties, electrical fatigue life, and tensile properties before and after electrified annealing.

Finally, the EFO test was conducted on the original and annealed wires. A scanning electron microscope (SEM, HITACHI SU-5000, HITACHI, Tokyo, Japan) was used to examine the surface characteristics of FAB, including its microstructure and hardness. To assess the heat-affected zone (HAZ) length, micro-hardness measurements were taken at 30 µm intervals, shown in Figure 1d, and continued until the hardness matched the original hardness of the wire. Additionally, energy-dispersive X-ray spectroscopy (EDS) was employed to analyze the elemental composition of the wire and FAB, verifying the solidification segregation effect.

## 3. Results and Discussion

### 3.1. Microstructure and Mechanical Properties

**Figure 2:**
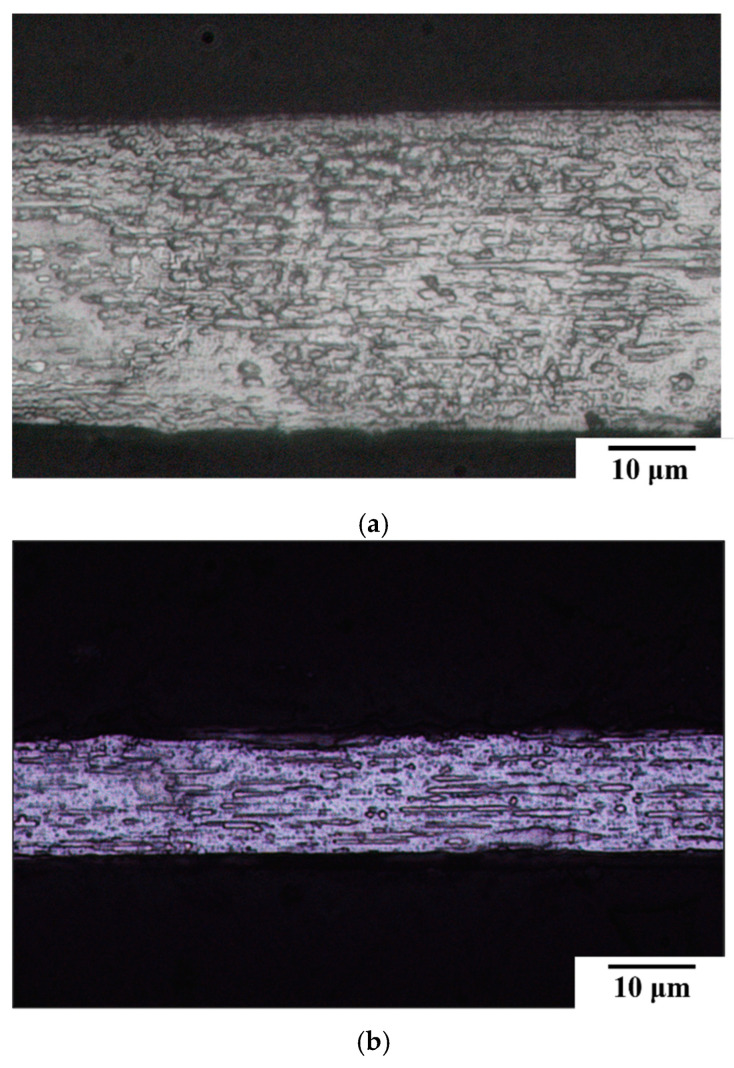

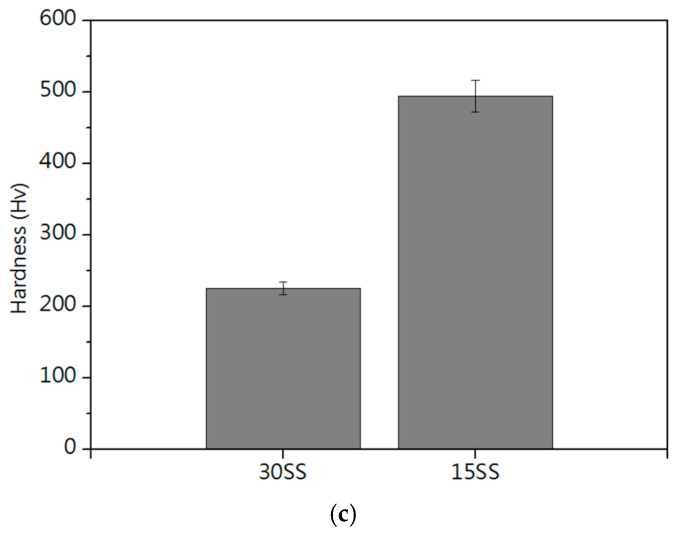
(**a**) Microstructure of 30SS; (**b**) microstructure of 15SS; and (**c**) micro-hardness of 30SS and 15SS.

Figure 2a,b present the microstructures of two 304L stainless steel fine wires with different diameters. Both exhibit elongated grains aligned with the drawing direction, a characteristic morphology induced by the cold drawing process. The internal structures of both wires are dense and free of noticeable defects. Figure 2c presents the hardness comparison, revealing that 15SS has a higher hardness than 30SS. This is primarily due to its smaller diameter, indicating a greater degree of cold working and a higher work-hardening effect. Consequently, 15SS is also expected to exhibit higher strength than 30SS.

**Figure 3:**
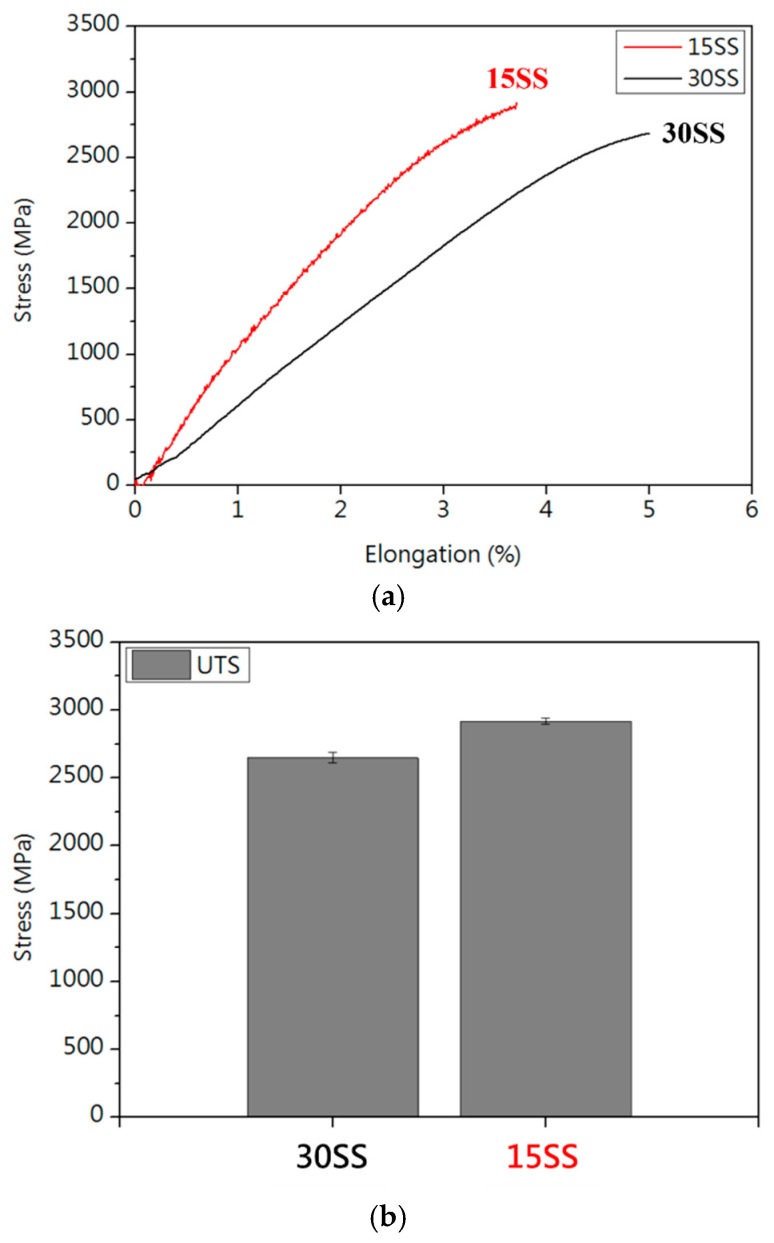

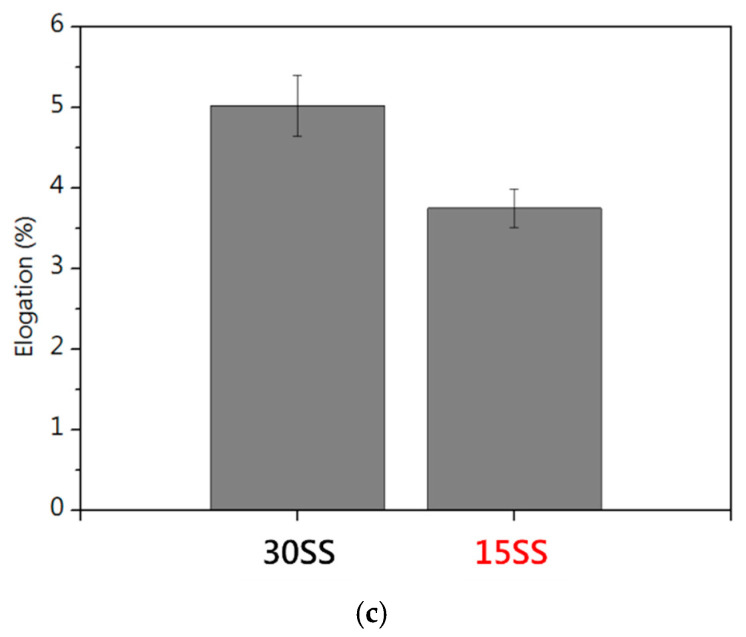
Tensile properties of 30SS and 15SS: (**a**) stress–strain curves; (**b**) tensile strength; and (**c**) elongation.

Figure 3 presents the tensile test results of both wires. Due to the higher work-hardening effect in 15SS, it exhibits higher tensile strength than 30SS, aligning with the predictions from Figure 2. Figure 3c shows that both wires have very low ductility, making them unsuitable for wire bonding applications. To address this issue, annealing is considered as a potential solution.

### 3.2. IV Curve and Electrical Fatigue Test

**Figure 4:**
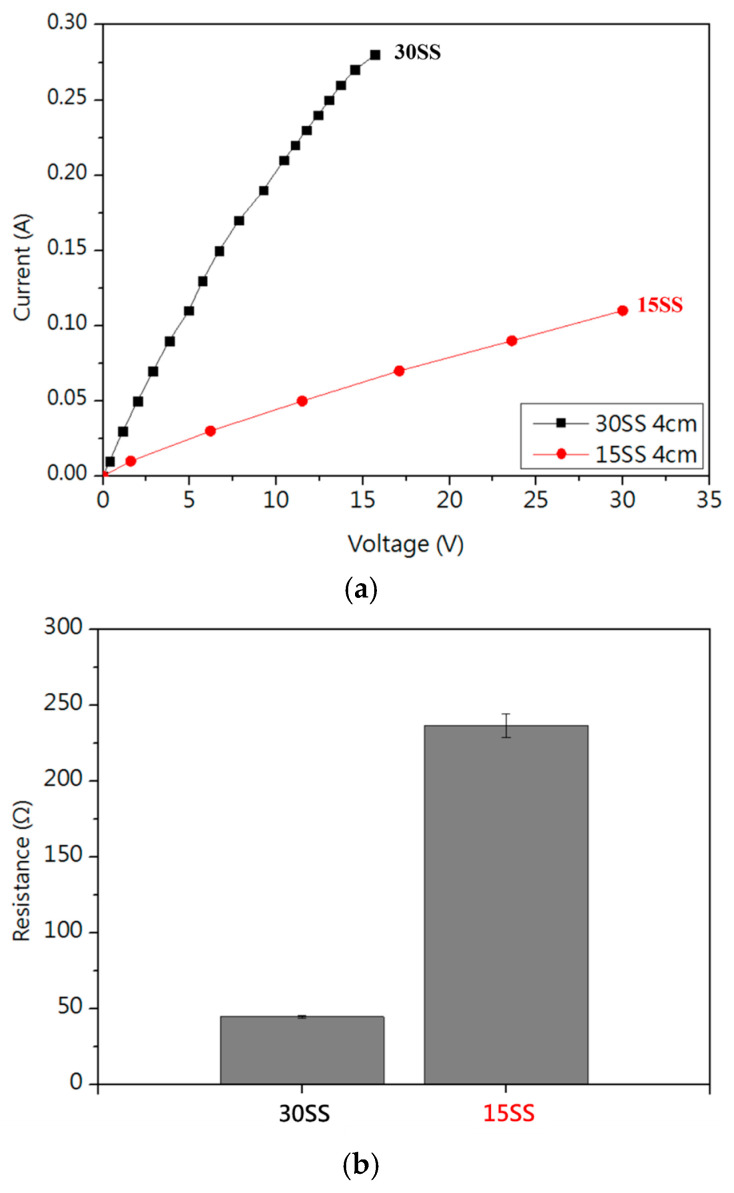
Electrical properties of 30SS and 15SS: (**a**) I-V curve; (**b**) resistance.

Figure 4a shows the I-V curve, where the dynamic resistance was measured in the linear region. Figure 4b shows that the resistance of 15SS is higher than that of 30SS, primarily due to its smaller cross-sectional area. Under the same applied voltage, this results in a higher resistance. The greater Joule heating effect induced by the higher resistance causes the fuse current (FC) of 15SS at a 4 cm length to be lower than that of 30SS, as shown in Table 1.

**Figure 5:**
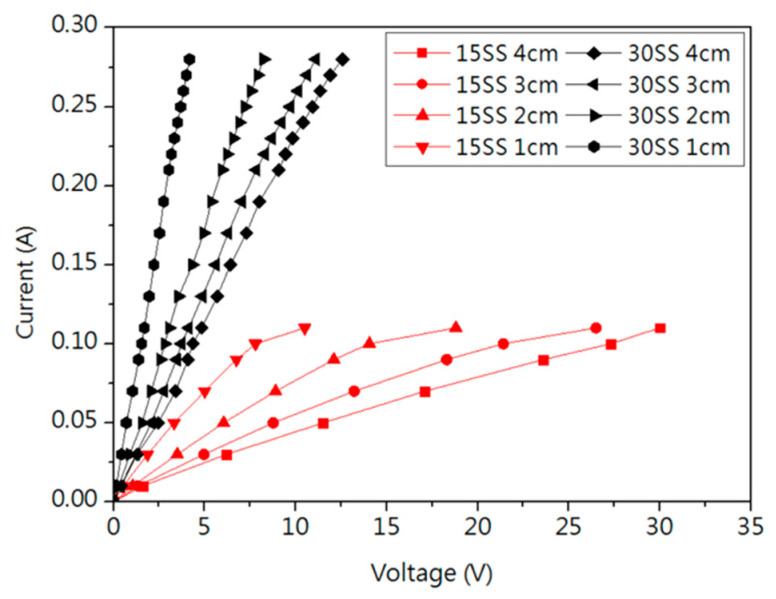
I-V Curve for 30SS and 15SS at different wire lengths.

Figure 5 presents the I-V curves of 15SS and 30SS measured at wire lengths ranging from 1 cm to 4 cm. It is observed that the fusing current remains unchanged despite the reduction in wire length. After converting the measurements into resistance values (Table 1), both 15SS and 30SS exhibit a significant decrease in resistance as the wire length shortens, with a notable difference between the 1 cm and 4 cm wires. Using the equation $R=\rho \times \frac{L}{A}$ (where R is resistance, ρ is resistivity, L is wire length, and A is the cross-sectional area), the calculated resistivities are $4.33\times 10^{-6}$ Ω·m for 15SS and $3.86\times 10^{-6}$ Ω·m for 30SS. When extrapolating to the wire bonding scale (100 µm), the resistance of both wires converges to values below 1 Ω.

**Figure 6:**
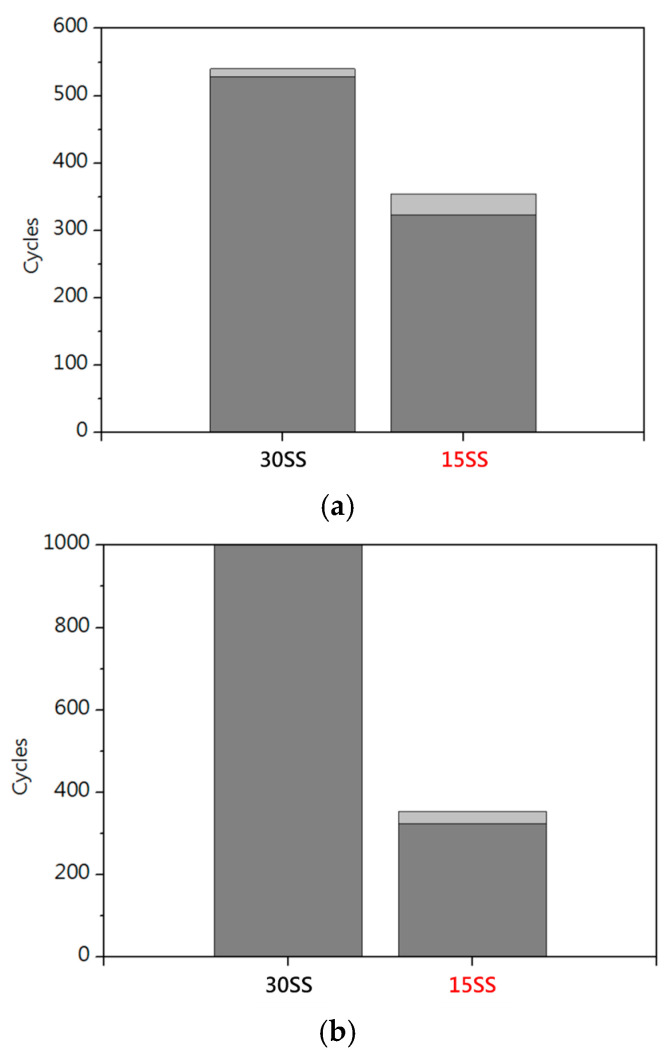
Electrical fatigue testing of 30SS and 15SS: (**a**) 80% fuse current; (**b**) 0.08 A.

Figure 6a shows the electrical fatigue tests conducted at 80% fuse current of each wire (15SS: 0.08 A, 30SS: 0.22 A). Figure 6b shows the electrical fatigue tests based on the 80% fuse current of 15SS (0.08 A). Due to its lower resistance, 30SS generates less Joule heating under long electrical loading, resulting in superior electrical fatigue life. Notably, both wires exhibit better electrical fatigue performance compared to Cu wires (30SS: 528 cycles/15SS: 339 cycles/Cu wire: 100 cycles) [14].

### 3.3. Microstructure and Micro-Hardness of Vacuum-Annealed Wires

**Figure 7:**
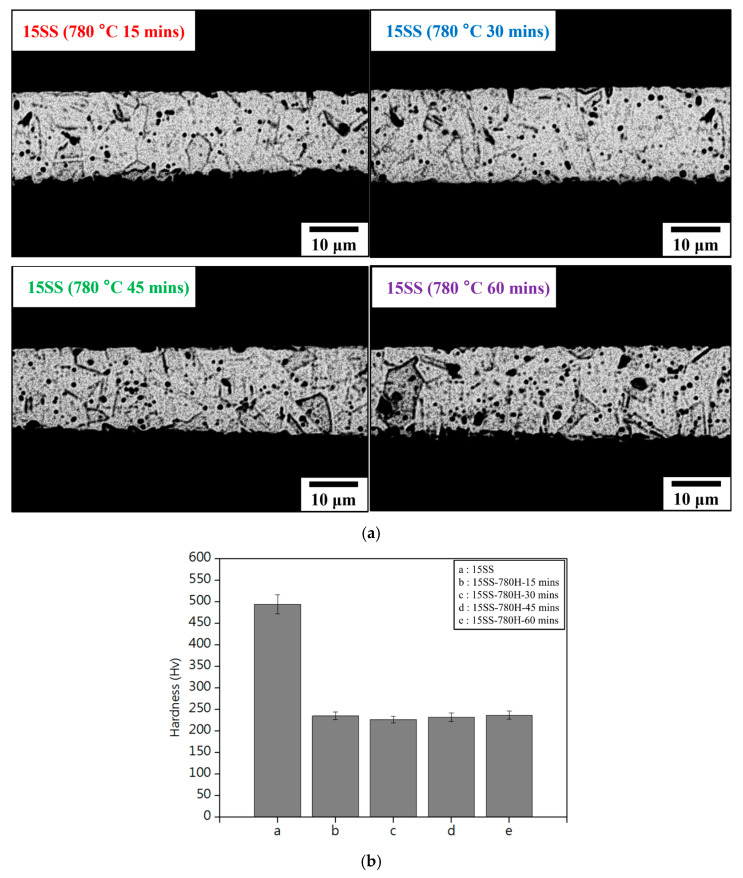
15SS-780H annealed for 15, 30, 45, and 60 min (**a**) microstructure; (**b**) micro-hardness.

Figure 7a presents the microstructure of 15SS after vacuum annealing at 780 °C for 15, 30, 45, and 60 min. The annealed wires (15SS-780H) exhibited more equiaxed grains with increased grain size, while the original elongated grain structure disappeared. Figure 7b compares the hardness of 15SS and 15SS-780H, showing that annealing at 780 °C for 15 min significantly reduced hardness from 494 Hv to 235 Hv. However, extending the holding time beyond 15 min did not result in further hardness reduction.

**Figure 8:**
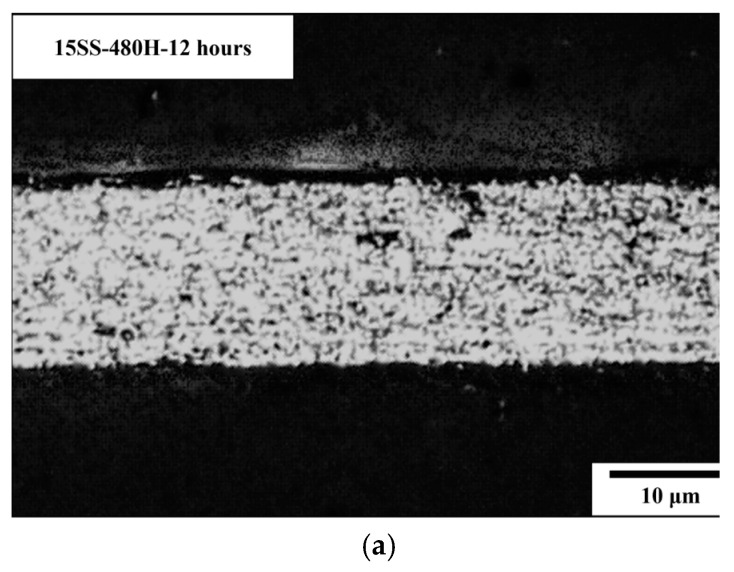

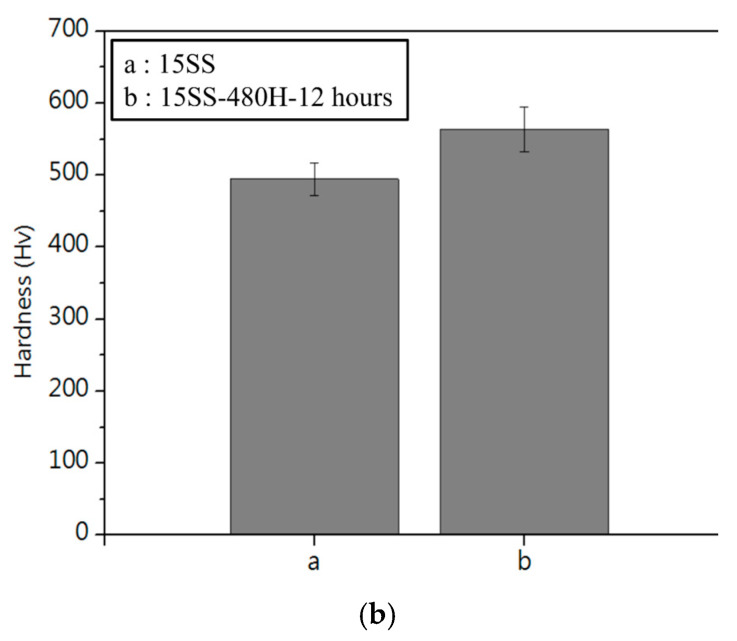
15SS-480H-12 h (**a**) microstructure; (**b**) micro-hardness.

Figure 8a presents the microstructure of 15SS after vacuum annealing at 480 °C for 12 h (15SS-480H-12 h). Figure 8b compares the hardness of 15SS and 15SS-480H-12 h, showing that annealing transformed the elongated grains into equiaxed grains. However, in contrast to 15SS-780H, the grain structure exhibited no significant changes after long-duration low-temperature annealing, and there was no observable reduction in hardness at 480 °C. In other words, in this study, 304L stainless steel wires annealing at 480 °C after work hardening do not induce recrystallization. This is attributed to the strain-induced martensitic transformation (SIMT) in austenitic stainless steel, where extensive cold working promotes martensite formation and high dislocation density, leading to increased hardness [15]. Therefore, a sufficiently high annealing temperature is required to transform martensite back into austenite.

**Figure 9:**
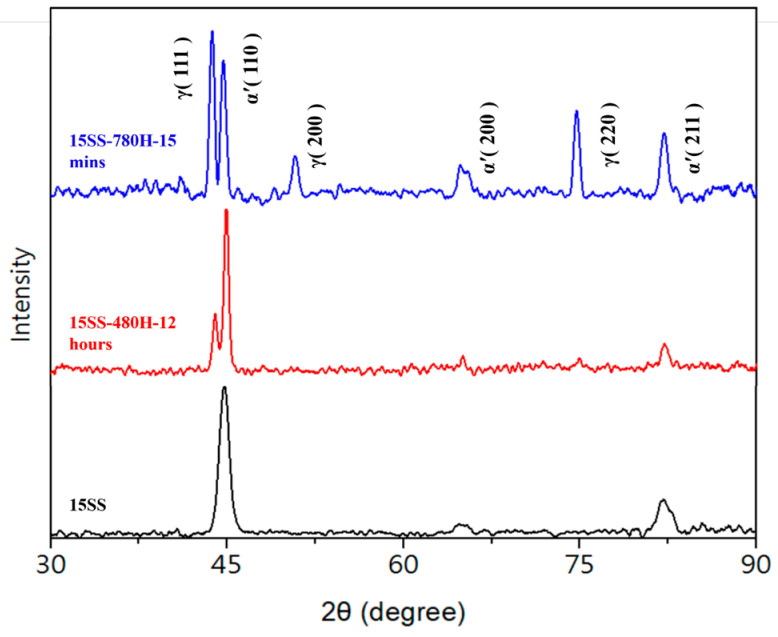
XRD analysis of the wires.

Additionally, the literature on 304 stainless steel indicates that after cold working, hardness increases, and annealing at 200 °C, 300 °C, or 450 °C fails to induce recrystallization or phase transformation, preventing any significant reduction in hardness (whereas conventional steels typically exhibit tempering softening) [16]. To verify this, X-ray diffraction (XRD) analysis was conducted on the original wire and the two vacuum-annealed wires. Figure 9 presents the XRD results of the three wire samples, revealing a significant presence of the martensite phase in 15SS. This aligns with the previous literature, which states that extensive cold working induces the SIMT phenomenon in austenitic stainless steel, leading to martensite formation (whereas conventional 304 stainless steel is fully austenitic). Notably, the martensite peak in 15SS-480H-12 hrs remains higher than the austenite peak, indicating that annealing at 480 °C is insufficient to revert martensite to austenite. The literature reports that annealing deformed 304 stainless steel at 500 °C results in a martensite content of up to 70 wt.%, which is consistent with the findings of 15SS-480H-12 hrs [17]. In contrast, the highest peak in 15SS-780H-15 min corresponds to the austenite phase, demonstrating that annealing at 780 °C effectively reverts martensite to austenite, leading to a reduction in hardness. Additionally, the literature suggests that annealing austenitic stainless steel at 800 °C can nearly eliminate deformation-induced martensite, corroborating these results [18]. Based on these findings, this study selects the 780 °C, 15 min annealing condition as a reference parameter for determining the required current in the subsequent in-line electrical annealing process.

### 3.4. Electrical-Induced Temperature [19]

**Figure 10:**
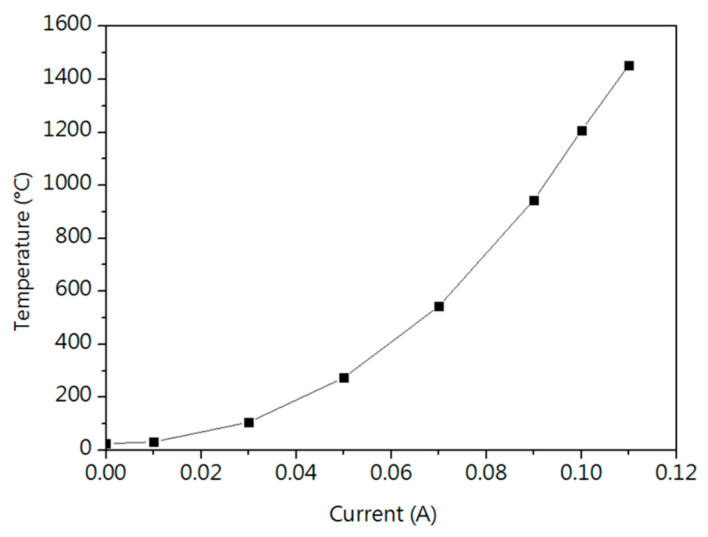
Relationship between current and induced temperature for 15SS.

Figure 10 shows the relationship between electrical current and induced temperature for 15SS, calculated by Equation (1).

(1) $$T=\frac{IV}{2.3085\times 10^{-3}}+25$$

The derivation is as follows: At thermal equilibrium, the heat dissipation of the wire equals the incoming electrical energy. This relationship can be expressed by Equation (2), where $J_{Q}$ represents the heat flux within the wire, A is the heat dissipation area, and k is the thermal conductivity. In the experiment, heat dissipates through two primary pathways: one is dissipating into the surrounding air ($J_{Q}^{air}$), the other is through conduction along the wire ($J_{Q}^{wire}$). The wire has a length (L) and a radius (r). It is assumed that the air temperature at a distance (3r) from the wire remains unaffected by the temperature of the wire, meaning the ambient air remains at room temperature. Additionally, the temperature gradient within the wire is assumed to reach its maximum at the center (L/2), causing the central region to heat up the fastest and reach the melting point of 304 stainless steel, ultimately leading to the fuse. Based on these assumptions, Equations (3) and (4) are derived, where Equation (3) represents the heat dissipation into the air, and Equation (4) accounts for the heat dissipation along the wire. The factor of 2 in Equation (4) accounts for heat loss from both ends of the wire. By combining Equations (2)–(5), the following is obtained:

*J_Q_*A = Ak∇T = IV (2)

(3) $$J_{Q}^{air}A=A_{\text{air}}k_{\text{air}}\nabla T_{\text{air}}$$

(4) $$J_{Q}^{wire}A={2A}_{\text{wire}}k_{\text{04SS}}\nabla T_{\text{wire}}$$

(A_air_ k_air_ ∇T_air_) + (2A_wire_ k_wire_ ∇T_wire_) = IV (5)

In the equations, k_air_ and k_wire_ represent the thermal conductivities of the air and wire, with values of 2.6 × 10^−2^ (W/Mk) and 16.3 (W/Mk), respectively. The surface area of the wire is defined as A_air = 2πrL, while its cross-sectional area is given by A_wire_ = πr^2^. The temperature gradients are expressed as ∇T_air_ = ∆T/3r and ∇ T_air_ = ∆T/0.5L, where ∆T represents the temperature difference between the wire and ambient air. By incorporating these definitions, Equation (5) is reformulated as follows:

2.3085 × 10^−3^ ∆T + 2.3044 × 10^−6^ ∆T = IV (6)

Since 2.3085 × 10^−3^ is significantly larger than 2.3044 × 10^−6^, the latter term can be neglected. Consequently, Equation (6) can be simplified as follows:

(7) $$T=\frac{IV}{2.3085\times 10^{-3}}+25$$

Using Equation (7), the relationship between electrical current and induced temperature for the 15SS wire can be determined. From this curve, the required current to reach 780 °C was calculated, serving as the basis for the subsequent electrifying annealing process.

### 3.5. Microstructure and Mechanical Properties of Electrically Annealed Wires

**Figure 11:**
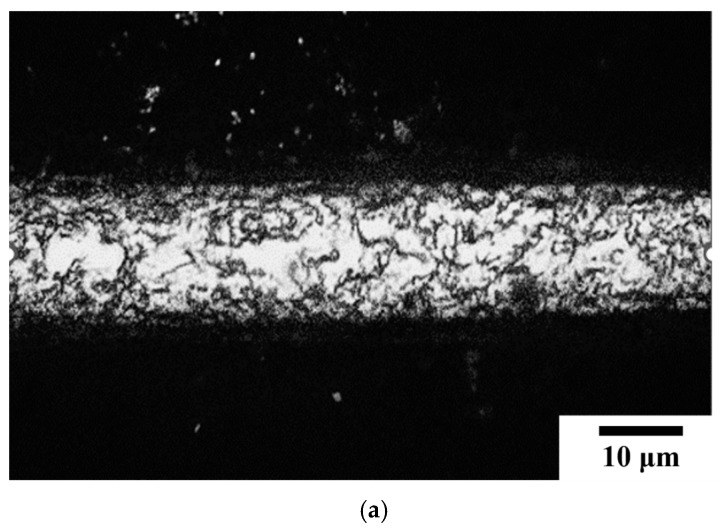

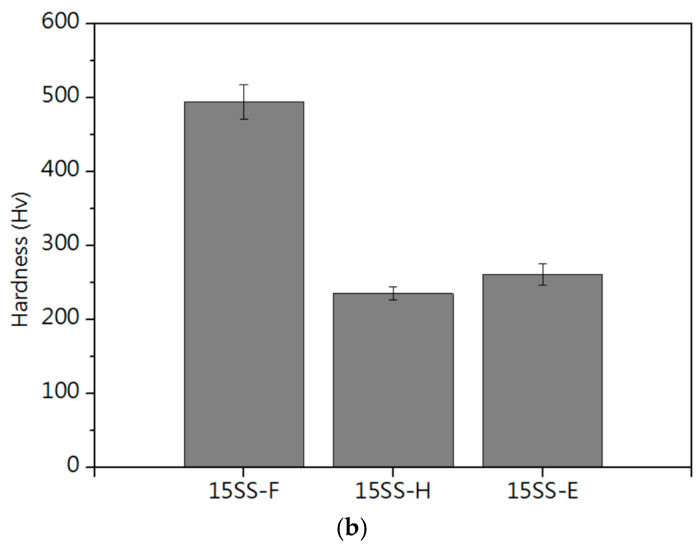
The 15SS-E (**a**) microstructure; (**b**) micro-hardness.

Figure 11a presents the microstructure of the wire undergoing an electrified annealing at 0.08 A for 15 min (15SS-E). The grain structure became more uniform, with an increase in grain size. This indicates that electrical annealing promotes grain growth, which is expected to reduce hardness. Figure 11b compares the hardness of the original wire (15SS-F), the vacuum-annealed wire at 780 °C for 15 min (15SS-H), and the electrically annealed wire (15SS-E). The results show that the hardness of 15SS-E decreases significantly from 494 Hv to 261 Hv and closely matches that of 15SS-H, demonstrating that electrical annealing achieves a softening effect comparable to conventional vacuum furnace annealing. This observation aligns with the conclusions drawn from Figure 11a.

**Figure 12:**
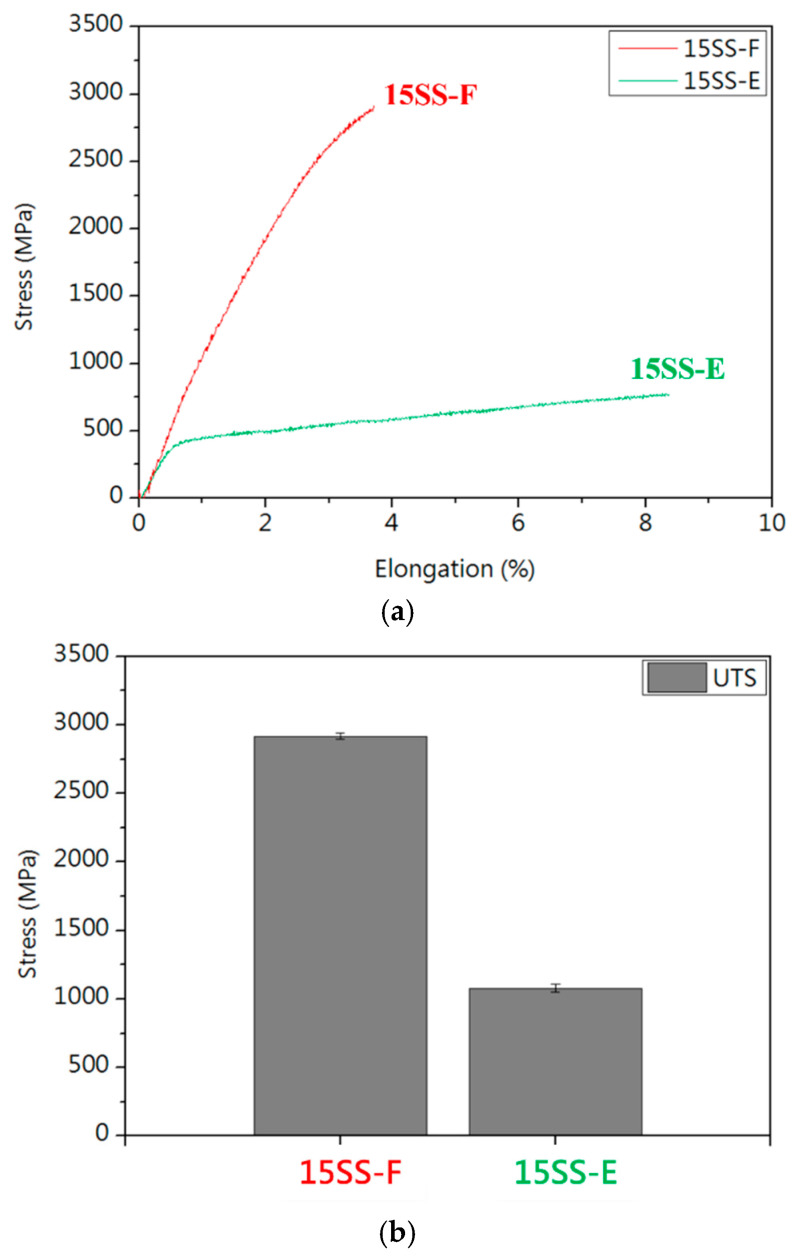

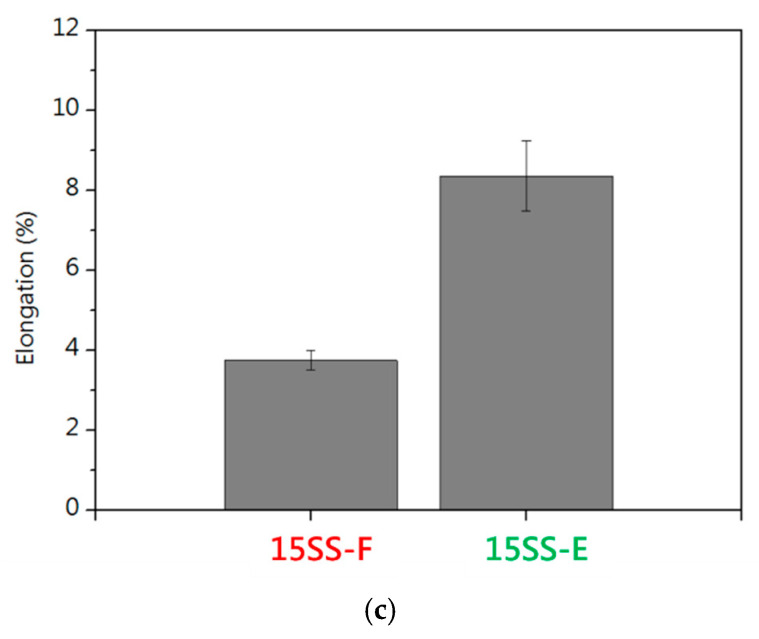
Tensile properties of 15SS-F and 15SS-E: (**a**) stress–strain curves; (**b**) tensile strength; and (**c**) elongation.

Figure 12 compares the tensile properties of 15SS-F and 15SS-E. After electrified annealing, the wire exhibited reduced strength and increased elongation. This softening effect addresses the excessive strength and limited elongation of the original 304 stainless steel fine wire (15SS-F), enhancing its suitability for wire bonding applications.

### 3.6. Electrical Properties of Electrically Annealed Wires

**Figure 13:**
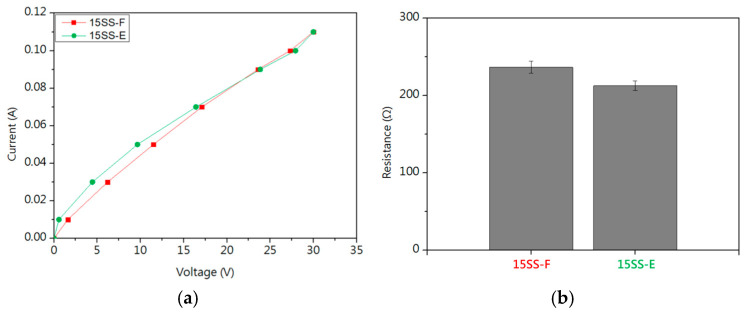
Electrical properties of 15SS-F and 15SS-E: (**a**) I-V curve; (**b**) resistance.

Figure 13a compares the I-V curves of 15SS-F and 15SS-E. According to the referenced literature, prolonged electrical conduction increases overall resistance due to Joule heating and the formation of an oxide layer on the surface [20]. Therefore, only the linear region where the current is below 0.05 A was used to calculate resistance (Figure 13b). The results indicate that the resistance of 15SS-E is lower than that of 15SS-F, attributed to grain growth and a reduction in grain boundary density after electrical annealing, which decreases resistance. The literature also states that an increase in grain size reduces wire strength and resistivity while enhancing conductivity, aligning well with the result in Figure 13b [21].

**Figure 14:**
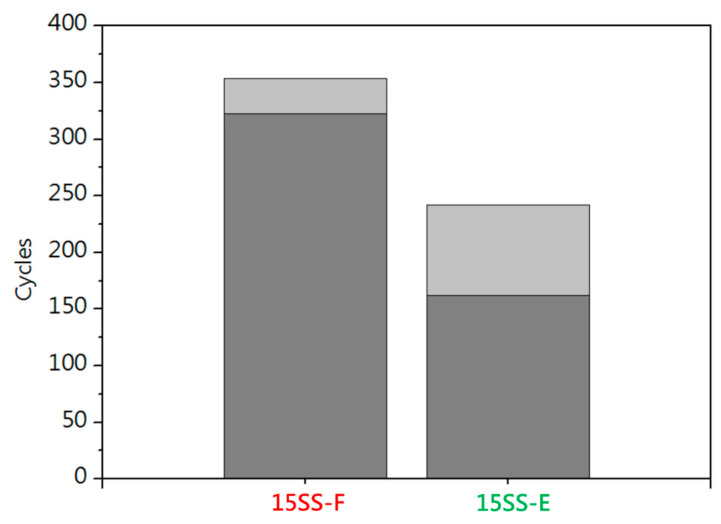
Electrical fatigue testing of 15SS-F and 15SS-E.

Figure 14 compares the electrical fatigue life of 15SS-F and 15SS-E, revealing a decline in fatigue performance for 15SS-E. This reduction can be attributed to two factors. One is the electrified annealing current (0.08 A), while the resistance of 15SS-E exceeded that of 15SS-F at currents above 0.05 A. As a result, the Joule heating effect during electrical fatigue testing was more pronounced, causing the wire temperature to rise more rapidly toward the fuse temperature. The other is the homogenization effect. 15SS-F underwent extensive cold working, accumulating a high level of stored energy, contributing to its superior electrical fatigue life of up to 300 cycles. During electrified annealing, part of this stored energy was released, promoting grain uniformity. Consequently, while 15SS-E exhibited lower strength and improved ductility, its electrical fatigue life was reduced.

### 3.7. Electronic Flame-Off (EFO) Test

**Figure 15:**
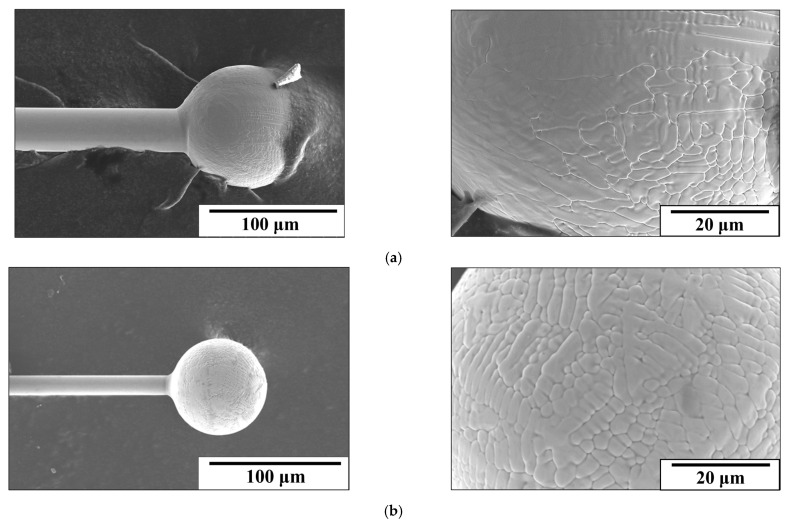
SEM images of ball surface morphology: (**a**) 30SS (**b**) 15SS-F.

**Figure 16:**
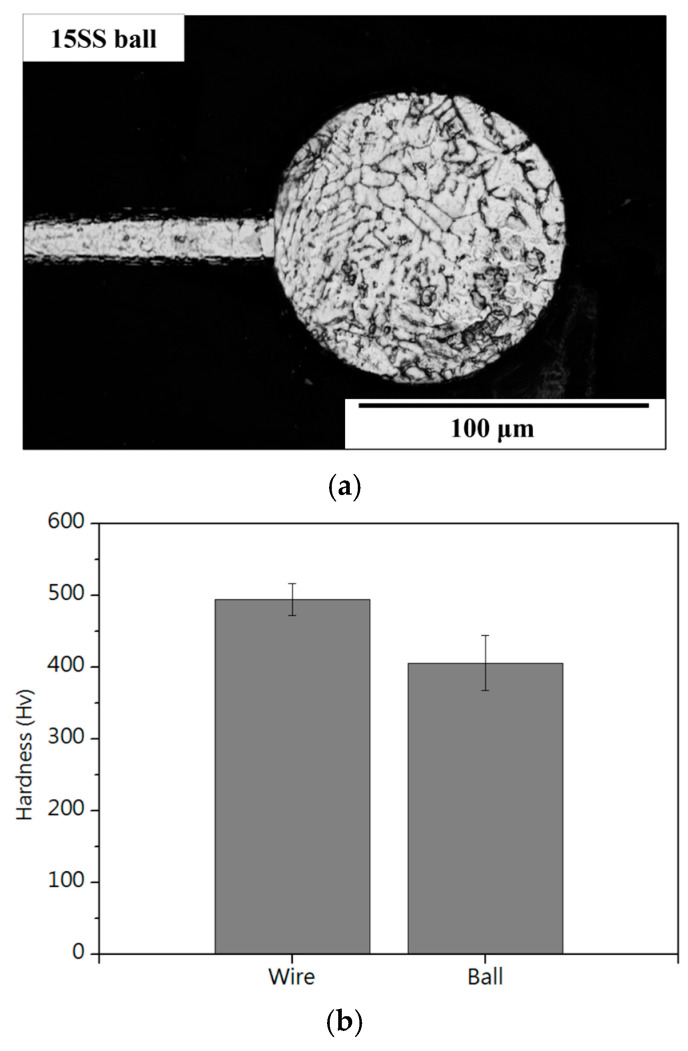
The 15SS-F (**a**) microstructure of FAB; (**b**) micro-hardness of FAB and wire.

Figure 15a,b present SEM images of 30SS and 15SS-F after the EFO process, confirming that 304 stainless steel fine wires can successfully undergo discharge ball formation. The images also reveal the presence of extensive dendritic structures on the surface of FAB. Figure 16a further illustrates that the ball microstructure consists of columnar and dendritic grain structures. According to the literature, the solidification behavior of 304 stainless steel is influenced by the Cr_eq_/Ni_eq_, which can be determined using the following equations: [22,23,24,25]

Ni_eq_ = [Ni] + 30[C] + 30[N] + 0.5[Mn] (8)

Cr_eq_ = [Cr] + [Mo] + 1.5[Si] + 0.5[Nb] (9)

In the equations, [A] represents wt.% of element A. Using Equations (8) and (9), the Cr_eq_/Ni_eq_ for 304 stainless steel is calculated to be 1.77, corresponding to an AF-mode solidification behavior. In AF-mode solidification, the surface of 304 stainless steel tends to form a skeletal ferrite structure. However, when the cooling rate during solidification exceeds a critical threshold, the solidification mode transitions to FA mode, where the surface preferentially precipitates the austenite phase, forming a dendritic grain structure.

**Figure 17:**
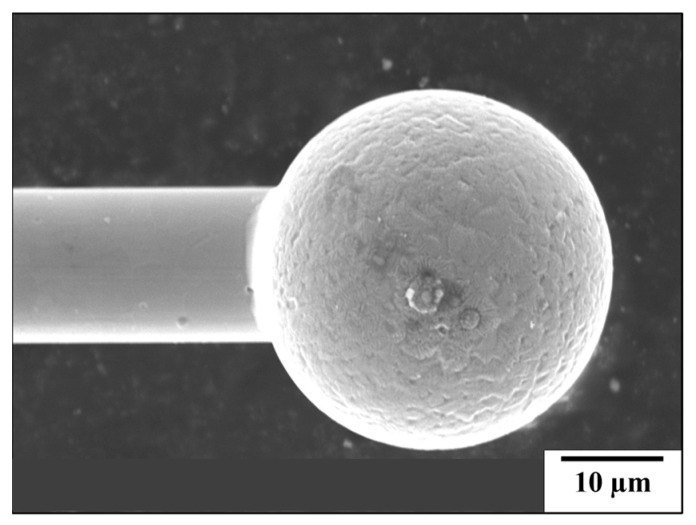
SEM images of ball surface morphology of 15SS-E.

**Figure 18:**
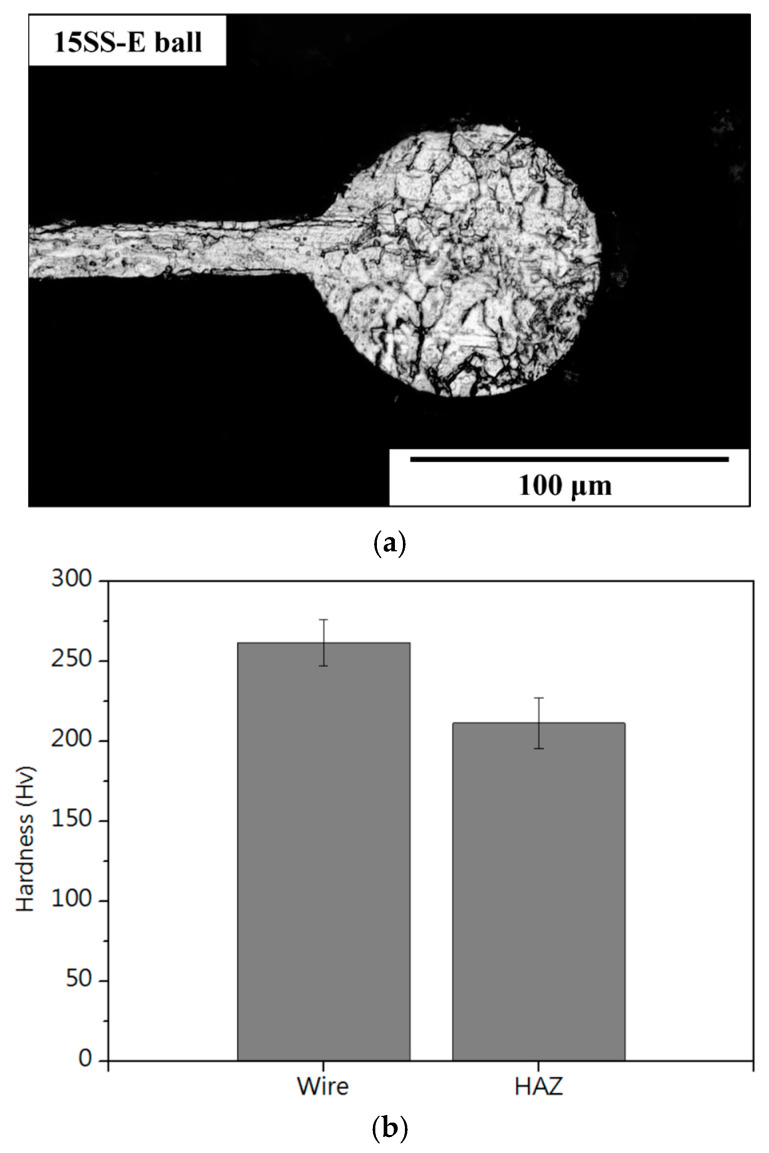
The 15SS-E (**a**) microstructure of FAB; (**b**) micro-hardness of FAB and wire.

**Figure 19:**
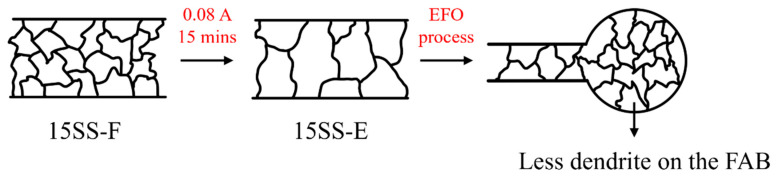
Illustration of electrically induced recrystallization and EFO process.

Notably, electrically annealed wires exhibit better ball formation characteristics. Figure 17 and Figure 18a show that after the EFO process, the surface and internal dendritic structures of the 15SS-E ball have largely disappeared. This is attributed to recrystallization during electrical annealing, which homogenizes the internal grain structure (Figure 19). This uniformity reduces compositional segregation during solidification and enhances thermal conductivity, thereby minimizing the formation of dendritic structures.

**Figure 20:**
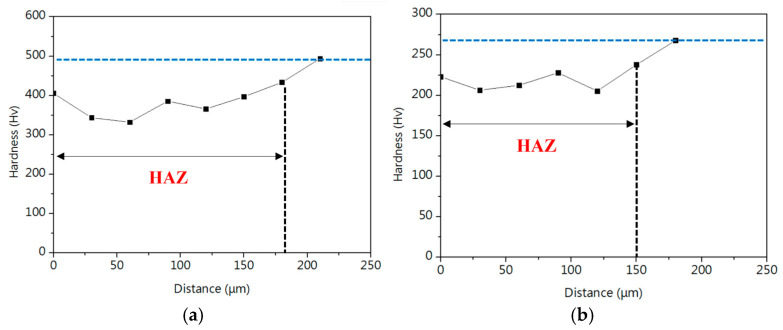
Length of HAZ (**a**) 15SS-F and (**b**) 15SS-E.

**Figure 21:**
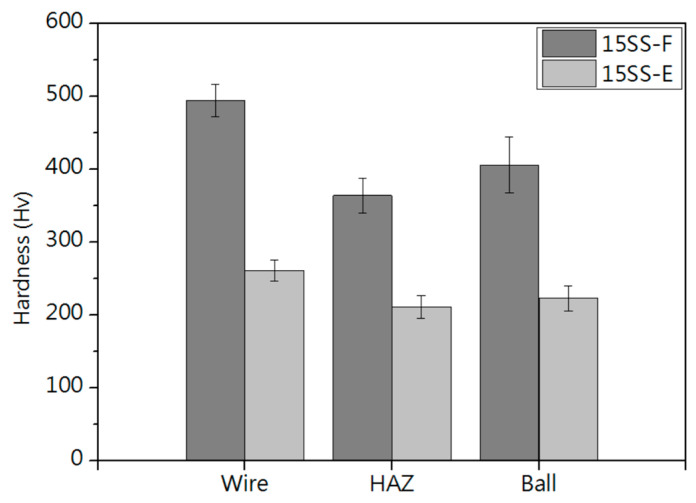
Comparison of micro-hardness in wire, HAZ, and FAB for 15SS-F and 15SS-E.

Figure 16b and Figure 18b compare the hardness of the two wires and their respective ball formations, while Figure 20a,b present hardness measurements in the heat-affected zone (HAZ). The results indicate that the HAZ length for the original wire (15SS-F) ranges from 180 to 210 µm, whereas, for the electrically annealed wire (15SS-E), it is reduced to 150–180 µm. By averaging the hardness values of the HAZ and comparing them with the wire and ball hardness (Figure 21), it was confirmed that both 15SS-F and 15SS-E exhibit higher hardness than the FAB and HAZ area. This is due to the high-temperature melting and rapid solidification during discharge ball formation, which results in a large-grained FAB and HAZ area. Additionally, after electrical annealing, the hardness of the wire, ball, and HAZ significantly decreased, further demonstrating the superior ball-forming characteristics of electrically annealed stainless steel fine wires.

**Figure 22:**
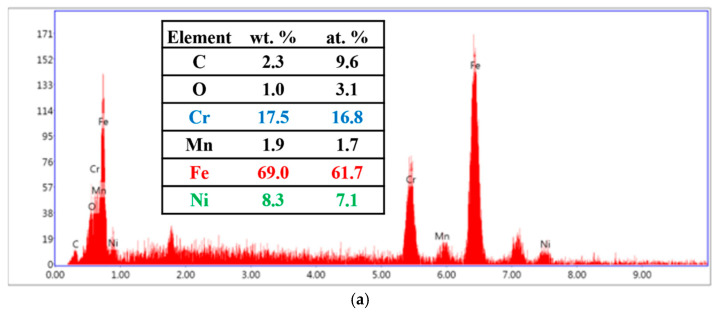

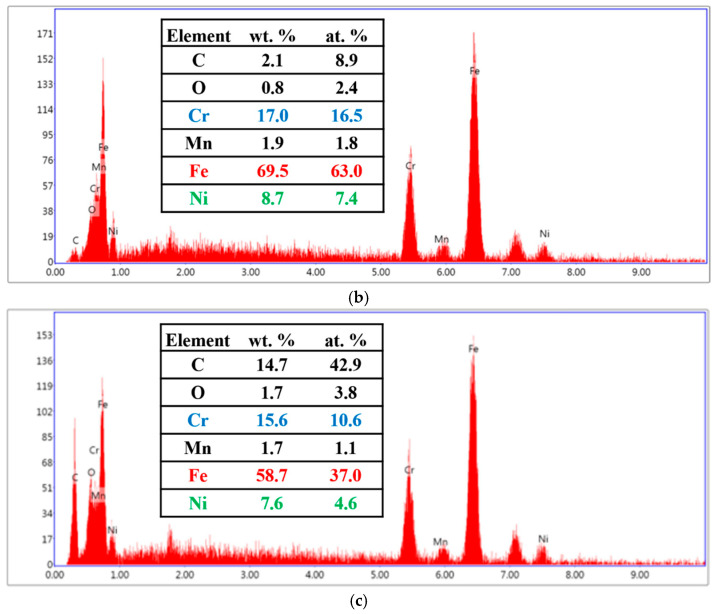
Elemental analysis of 15SS-F: (**a**) wire; (**b**) HAZ; and (**c**) FAB.

**Figure 23:**
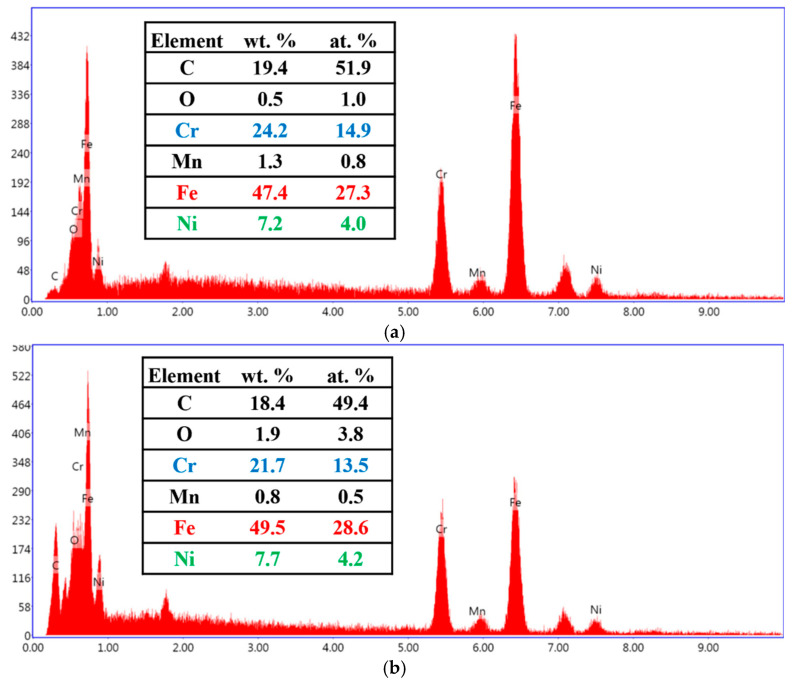

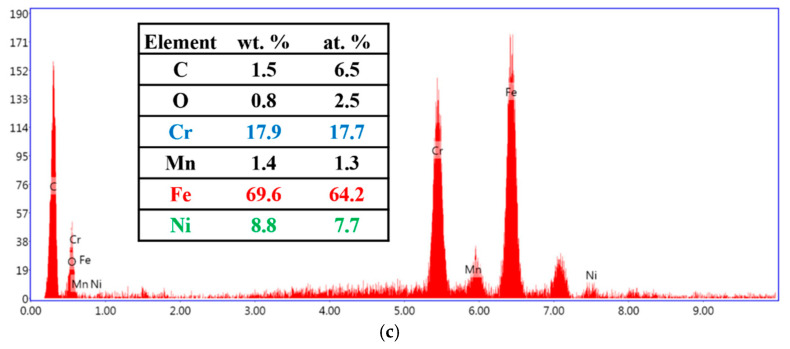
Elemental analysis of 15SS-E: (**a**) wire; (**b**) HAZ; and (**c**) FAB.

Figure 22 and Figure 23 present EDS analyses of the wire, HAZ, and FAB surface after ball formation for 15SS-F and 15SS-E. The results confirm that the primary elemental composition of the HAZ, neck region, and ball remains consistent with the original wire, indicating that electrically annealed wires maintain their feasibility for wire bonding applications.

## 4. Conclusions

(1) Due to the cold drawing process, the grains of the 304L stainless steel fine wire are elongated along the drawing direction, forming an elongated grain structure. The material exhibits a dense, defect-free internal structure with significant work hardening effects, resulting in excellent strength (2916 MPa) and hardness (494 Hv).
(2) Both 30 µm and 15 µm 304L stainless steel fine wires exhibit excellent electrical fatigue resistance and successfully undergo the EFO process, reaching the requirements for wire bonding applications.
(3) Vacuum annealing at 480 °C was ineffective in reversing the SIMT-induced martensite back to austenite in the wire, resulting in no reduction in hardness. In contrast, annealing at 780 °C successfully facilitated the reverse transformation of martensite in 304L stainless steel wire, decreasing strength and hardness. By converting the 780 °C condition to an equivalent electrical annealing current of 0.08 A, the electrically annealed wire exhibited softening and enhanced ductility, making it more suitable for wire bonding applications.
(4) Cold-drawn 304L stainless steel fine wires develop a dendritic crystal structure after the EFO process. In contrast, electrified annealing reduces solidification segregation in the ball microstructure, minimizes the heat-affected zone, and decreases the number of dendritic structures, enhancing their suitability for wire bonding applications.

## Figures and Tables

**Table 1:** Fuse current and resistance for 15SS and 30SS.

	15SS	30SS
FC at 4 cm (A)	0.11	0.28
Resistance at 4 cm (Ω)	236.46	44.85
Resistance at 3 cm (Ω)	177.17	40.83
Resistance at 2 cm (Ω)	120.72	30.16
Resistance at 1 cm (Ω)	66.77	14.75
Resistance at 100 µm (Ω)	0.61	0.14

## Data Availability

The original contributions presented in the study are included in the article, further inquiries can be directed to the corresponding author.

## References

[1] Chen W.T., Chang C.S., Charsky R.S. An overview of electrical and mechanical aspects of electronic packaging. Proceedings of the 1990 IEEE International Symposium on Circuits and Systems (ISCAS).

[2] Hu G. Comparison of copper, silver and gold wire bonding on interconnect metallization. Proceedings of the 2012 13th International Conference on Electronic Packaging Technology & High Density Packaging.

[3] Zhou H., Zhang Y., Cao J., Su C., Li C., Chang A., An B. (2023). Research progress on bonding wire for microelectronic packaging. Micromachines.

[4] Jaafar N.B., Ching E.W.L. Comparison of Au/Al, Cu/Al and Ag/Al in wirebonding assembly and IMC growth behavior. Proceedings of the 2016 IEEE 18th Electronics Packaging Technology Conference (EPTC).

[5] Lu C.T. The challenges of copper wire bonding. Proceedings of the 2010 5th International Microsystems Packaging Assembly and Circuits Technology Conference.

[6] George G., Shaikh H. (2002). Introduction to austenitic stainless steels. Corrosion of Austenitic Stainless Steels.

[7] Tuthill A.H., Covert R.A. (2000). Stainless steels: An introduction to their metallurgy and corrosion resistance. Dairy Food Environ. Sanit..

[8] Xu Q., Zhu J., Zong Y., Liu L., Zhu X., Zhang F., Luan B. (2021). Effect of drawing and annealing on the microstructure and mechanical properties of 304 austenitic stainless steel wire. Mater. Res. Express.

[9] Milad M., Zreiba N., Elhalouani F., Baradai C. (2008). The effect of cold work on structure and properties of AISI 304 stainless steel. J. Mater. Process. Technol..

[10] Pham H.T., Iwamoto T. (2018). An evaluation of fracture properties of type-304 austenitic stainless steel at high deformation rate using the small punch test. Int. J. Mech. Sci..

[11] Prasad K.S., Rao C.S., Rao D.N. (2014). A review on welding of AISI 304L austenitic stainless steel. J. Manuf. Sci. Prod..

[12] Chuang H.C., Hung F.Y., Lui T.S., Chen L.H. (2011). Effect of the twins on mechanical properties of AISI 304 stainless steel wire using electrical current method. Mater. Trans..

[13] Panov D., Kudryavtsev E., Chernichenko R., Smirnov A., Stepanov N., Simonov Y., Zherebtsov S., Salishchev G. (2021). Mechanisms of the reverse martensite-to-austenite transformation in a metastable austenitic stainless steel. Metals.

[14] Chang Y.T., Hung F.Y., Wu B.D. (2024). Comparison of the Microstructural Characteristics and the Electrothermal Fracture Mechanism of Au-Pd-Coated Copper Wire and Cu-Ti Micro-alloyed Wire. J. Electron. Mater..

[15] Choi J.Y., Jin W. (1997). Strain induced martensite formation and its effect on strain hardening behavior in the cold drawn 304 austenitic stainless steels. Scr. Mater..

[16] Lee S.H., Lee J.C., Choi J.Y., Nam W.J. (2010). Effects of deformation strain and aging temperature on strain aging behavior in a 304 stainless steel. Met. Mater. Int..

[17] Salgado J., Fava J., Spinosa C. (2024). A study of the reverse martensitic transformations in austenitic stainless steels by calorimetry and isothermal heat treatments. Matéria.

[18] Sohrabi M.J., Naghizadeh M., Mirzadeh H. (2020). Deformation-induced martensite in austenitic stainless steels: A review. Arch. Civ. Mech. Eng..

[19] Balluffi R.W., Allen S.M., Carter W.C. (2005). Kinetics of Materials.

[20] Wu B.D., Hsu C.K., Huang B.C., Hung F.Y. (2024). Effect of electro-thermal diffusion induced deterioration on the failure mechanism of Pd-Au-coated Cu wires. J. Mater. Sci. Mater. Electron..

[21] Hou J., Sun P., Wang Q., Zhang Z., Zhang Z. (2022). Breaking the trade-off relation between strength and electrical conductivity: Heterogeneous grain design. Acta Metall. Sin..

[22] Fu J.W., Yang Y.S., Guo J.J., Tong W.H. (2008). Effect of cooling rate on solidification microstructures in AISI 304 stainless steel. Mater. Sci. Technol..

[23] Ma J.C., Yang Y.S., Tong W.H., Fang Y., Yu Y., Hu Z.Q. (2007). Microstructural evolution in AISI 304 stainless steel during directional solidification and subsequent solid-state transformation. Mater. Sci. Eng. A.

[24] Fu J.W., Yang Y.S., Guo J.J., Ma J.C., Tong W.H. (2009). Microstructure evolution in AISI 304 stainless steel during near rapid directional solidification. Mater. Sci. Technol..

[25] Huang F., Wang X., Zhang J., Ji C., Yuan F., Yan Y.U. (2008). In situ observation of solidification process of AISI 304 austenitic stainless steel. J. Iron Steel Res. Int..

